# Charge deformation and orbital hybridization: intrinsic mechanisms on tunable chromaticity of Y_3_Al_5_O_12_:Ce^3+^ luminescence by doping Gd^3+^ for warm white LEDs

**DOI:** 10.1038/srep11514

**Published:** 2015-07-15

**Authors:** Lei Chen, Xiuling Chen, Fayong Liu, Haohong Chen, Hui Wang, Erlong Zhao, Yang Jiang, Ting-Shan Chan, Chia-Hsin Wang, Wenhua Zhang, Yu Wang, Shifu Chen

**Affiliations:** 1 School of Materials Science and Engineering, Hefei University of Technology, Hefei 230009, China; 2 Shanghai Institute of Ceramics, Chinese Academy of Sciences, Shanghai 200050, China; 3 National Synchrotron Radiation Research Centre, Hsinchu 30076, Taiwan; 4 National Synchrotron Radiation Laboratory, University of Science and Technology of China, Hefei 230026, China; 5 Shanghai Synchrotron Radiation Facilities, Shanghai Institute of Applied Physics, Chinese Academy of Sciences, Shanghai 201204, China; 6 Department of Chemistry, Anhui Science and Technology University, Fengyang 233100, China

## Abstract

The deficiency of Y_3_Al_5_O_12_:Ce (YAG:Ce) luminescence in red component can be compensated by doping Gd^3+^, thus lead to it being widely used for packaging warm white light-emitting diode devices. This article presents a systematic study on the photoluminescence properties, crystal structures and electronic band structures of (Y_1−x_Gd_x_)_3_Al_5_O_12_: Ce^3+^ using powerful experimental techniques of thermally stimulated luminescence, X-ray diffraction, X-ray absorption near edge structure (XANES), extended X-ray absorption fine structure (EXAFS) and ultraviolet photoelectron spectra (UPS) of the valence band, assisted with theoretical calculations on the band structure, density of states (DOS), and charge deformation density (CDD). A new interpretation from the viewpoint of compression deformation of electron cloud in a rigid structure by combining orbital hybridization with solid-state energy band theory together is put forward to illustrate the intrinsic mechanisms that cause the emission spectral shift, thermal quenching, and luminescence intensity decrease of YAG: Ce upon substitution of Y^3+^ by Gd^3+^, which are out of the explanation of the classic configuration coordinate model. The results indicate that in a rigid structure, the charge deformation provides an efficient way to tune chromaticity, but the band gaps and crystal defects must be controlled by comprehensively accounting for luminescence thermal stability and efficiency.

The Nobel Prize in Physics 2014 was awarded jointly to three scientists, Isamu Akasaki, Hiroshi Amano and Shuji Nakamura, to honour their significant invention of blue light-emitting diodes (LEDs), which enabled bright and energy-saving light sources^1^. White LEDs, a type of solid semiconductor device, exhibiting inherent superiorities over traditional incandescent and fluorescent lamps in terms of high efficiency, low-energy consumption, eco-friendliness without mercury pollution, long lifetime, and chip-proof solid-state encapsulation within a small volume, revolutionized human lighting after Edison’s invention^2^,^3^,^4^. However, almost all white LEDs consist of a blue LED chip combined with one or more luminescent materials, which convert part of the blue light into other wavelengths^2^,^3^,^4^. Among the commercially available phosphors, the yellow-emitting Ce^3+^ activated yttrium garnet, i.e., Y_3_Al_5_O_12_: Ce (YAG: Ce), is the most commonly used one.

Y_3_Al_5_O_12_ has a cubic garnet structure, generally expressed with the formula A_3_B_2_(CO_4_)_3_, where the Y (A) occupies a dodecahedral site coordinated with eightfold oxygen atoms, two of the five Al (B) atoms occupy octahedral sites, and the other three Al (C) occupy tetrahedral sites^4^,^5^,^6^,^7^,^8^. The excellent performance of YAG in terms of high rigidity, low thermal-expansion, high-optical transparency, high threshold for optical damage, and chemical inertness makes it applicable for a wide variety of applications. YAG is a member of the large family of garnet-structure compounds, including Andradite, Goldmanite, Grossular, Toturite, Uvarovite, etc. There has been a rich history of the discovery and usage of garnets. The earliest application dates back to the Bronze Age, when they were used as gemstones and abrasives^4^,^5^,^6^,^7^,^8^. The synthetic YAG single crystal was used as a diamond simulant until recently, due to its fairly high hardness and relatively high refractive index.

The scientific research on YAG has been active since 1928 when Menzer first determined the garnet structure had the space group Ia3d^4^,^5^,^6^,^7^,^8^. In 1964 at Bell Laboratories, Geusic demonstrated a type of solid-state laser that adopted YAG as the host^9^. Since then, the YAG: Nd and YAG: Er solid-state lasers have been widely used in medical surgery, optical communications, coherent laser radar, meteorology, and industrial manufacturing. The parity-allowed f-d transition of Ce^3+^ in YAG has a very short lifetime and high luminescence efficiency. In 1967, Blasse and Bril firstly reported YAG: Ce as a new phosphor for flying-spot cathode ray tubes^10^. Due to high light yield (20000 photons/ MeV) and fast response time, in the 1990s YAG: Ce and the higher density analogue Lu_3_Al_5_O_12_: Ce (LuAG) and (Lu,Gd)_3_(Al,Ga)_5_O_12_ were proposed as scintillators for X-ray and gamma-ray radiation detection, used in X-ray computed tomographic scanner (CTS) and positron emission tomography (PET) for medical imaging and in scanning electron microscopy (SEM)^10^,^11^,^12^,^13^,^14^.

Ce^3+^-activated YAG has been intensively studied with a renewed interest for applications in white LEDs^4^,^15^,^16^,^17^,^18^, since it was first introduced into white LEDs by the Nichia Corporation in 1994. Nearly all the high-power white LEDs that are used in street and home lighting are fabricated using YAG: Ce as a phosphor. In addition, the same garnet structure LuAG: Ce was used a green phosphor for white LEDs. Quite sensibly, manufacturers and many scholars pay close attention to YAG: Ce. In addition to efficiency, the chromaticity and thermal stability of the luminescence of the phosphor are crucial to white LED applications because changes in the emission colour and flux during operation as a result of heat released continuously from the p-n junction of LED chips are highly undesirable^4^,^15^,^19^. This type of white light produced by mixing the yellow from YAG: Ce and the blue (450–460 nm) from InGaN chips, exhibits a cold colour temperature in addition to poor colour rendering ability^15^,^16^,^17^,^18^,^19^,^20^,^21^,^22^, due to its deficiency in the red component in its emission spectrum. However, warm white is generally desired for indoor lighting. To decrease the colour temperature, an appropriate amount of Gd^3+^ usually is doped into YAG: Ce, the emission peak of which can be regulated from approximately 543 nm to maximum 575 nm by tailoring the Gd^3+^ concentration until the collapse of crystal lattice^15^,^16^,^17^,^18^,^19^,^20^,^21^,^22^. Nevertheless, the luminescence efficiency decreases and the thermal stability of luminescence deteriorate after doping Gd^3+^ into YAG: Ce. However, the understanding of the mechanisms involved in these processes is limited^4^,^15^.

Pan attributed the red shift of YAG: Ce emission upon Gd^3+^ substitution to the difference in ionic radius between Y^3+^ (0.89 Å) and Gd^3+^ (0.94 Å), based on a strong crystal field splitting due to the lattice expansion^23^. However, this is not consistent with the opposite behavior that is commonly reported in the case of an expansion of a host lattice, which usually gives rise to a decrease of the crystal field splitting and results in the shift of 5d–4f emission towards short wavelengths^24^. Robbin observed an interesting phenomenon regarding the luminescence of YAG: Ce; i.e., the characteristic absorption band of YAG: Ce near 460 nm decreases in intensity, whereas the band near 340 nm increases along with a temperature increase^25^. Chiang and Bachmam *et al.* provided an explanation about the worsening thermal luminescence quenching of YAG: Ce upon substitution of Y^3+^ by Gd^3+^ using a quantum mechanically based configurational coordinate model, hypothesizing that the garnet structure becomes soft after incorporating Gd^3+^ into the YAG lattice^21^,^25^. Through analysis of atomic displacement parameters (ADPs) from X-ray and neutron scattering and the evaluation of mean-square relative displacement parameters from extended X-ray absorption fine structure (EXAFS), Ram *et al.* confirmed that YAG has a very rigid crystal lattice with a high Debye temperature (θ_D_, an estimate of the temperature at which all vibration modes of a crystal are activated)^15^. According to Ram’s result, we can reasonably conclude that the YAG structure should become more rigid before it collapses into orthorhombic YAlO_3_ (YAP) when Y^3+^ is substituted by Gd^3+^, because Gd^3+^ has a larger radius in comparison with Y^3+^ and thus it will suffer intensive compression from other neighbouring atoms in the rigid structure[Fig f1][Fig f2][Fig f3][Fig f4][Fig f5][Fig f6].

Moreover, there are two facts that cannot be explained with the traditional configuration coordinate diagram. First, the soft structure indicates a large Stokes’ shift. Accordingly, the nonradiative energy loss with electrons relaxed from the 5d excited state to the 4f ground state through the crossover of their potential parabolas should increase with increasing Gd^3+^. However, the overlap of the normalized excitation and emission spectra of (Y_1−x_Gd_x_)_3_Al_5_O_12_: Ce^3+^ decreases with an increase of Gd^3+^ content in x value (as seen below in Fig. 7), indicating that the nonradiative transition does not increase. Second, the thermal vibration of the crystal lattice strongly depends on the weight of the atoms, and a heavy weight is helpful resisting thermal vibration. To this end, the thermal stability of YAG: Ce should be improved after doping with Gd^3+^ because of its heavier weight compared with Y^3+^. Practically, this result is not observed. Hence, some mechanisms should exist that play a more important role than the thermal vibration of the crystal lattice and are beyond the range of what the configuration coordinate theory can explain.

Dorenbos set forth that the ionization of an electron from the excited state to the conduction band is the genuine mechanism of thermal quenching of Eu^2+,^^26^. Ce^3+^ has a similar electronic configuration to Eu^2+^. Moreover, the temperature-dependent lifetime luminescence measurements for low Ce concentrations show that the intrinsic quenching temperature of Ce is higher than 700 K. Meijerink *et al.* attributed the low quenching temperature of the commercial YAG: Ce phosphor reported previously to thermally activated concentration quenching^4^. With respect to a further mechanism that causes the thermally activated concentration quenching of YAG: Ce, it was explained by energy migration to defects, but the types of defects that quench luminescence were not uncovered in reference^4^. Robbins also pointed out that “the nature of this quenching is not known, but it may involve energy loss to certain persistent but unidentified defect centres”^25^.

The anti-site defects, the oxidation of Ce^3+^ to Ce^4+^, and the ionized defects caused by the X-ray or gamma-ray radiation have been investigated in the single crystal scintillator of YAG: Ce. More than ten traps in YAG: Ce were reported by Zych^27^. However, the synthesis temperature of the YAG: Ce phosphor is not as high as the growth temperature of the YAG single crystal scintillator. Moreover, the energy of X-ray and gamma-ray radiation used in scintillators is far higher than the 460 nm photons emitted from LED chips, and will induce more defects. So, the defects that appear in the scintillator will not necessarily occur in the YAG: Ce phosphor^4^. In this work, crystal defects and trap depths were identified using the thermally stimulated luminescence. The crystal and electronic structure of (Y_1−x_Gd_x_)_3_Al_5_O_12_: Ce^3+^ were investigated using a combination of powerful experimental techniques, assisted with theoretical calculations. The results show that band gap and the energy barrier of Ce^3+^ ionization from the 5d_2_ state to the conduction band decreases with an increase of Gd^3+^ concentration.

## Experimental

The phosphors, (Y_1−x_Gd_x_)_3_Al_5_O_12_: Ce^3+^ (x = 0, 0.1, 0.3, 0.5, 0.7, 0.9 and 1.0), were synthesized with a solid-state reaction at 1500 °C for 6 hours in a 25%H_2_ + 75%N_2_ reduction atmosphere. 2.5% BaF_2_ and 2% H_3_BO_3_ in weight percent were used as fluxes. After reaction at high temperature, the fired products were ground and washed in water several times to remove the residual fluxes. Emission and excitation spectra were collected using a Hitachi F-4600 spectrometer. The thermal stability of the luminescence was examined using the spectrometer in combination with a heating apparatus, operated in an air environment. The phosphor was filled into a bronze sample cell. After heating to an objective temperature and keeping balance for 5 minutes, the emission and excitation spectra were recorded. The heating rate is less than 1 °C/ min and the precision of temperature control is less than 0.1 °C. The diffuse reflection spectra were collected using a UV-VIS-NIR spectrophotometer (Shimadzu, UV-3600). The thermally stimulated luminescence (TSL) was measured using a thermoluminescence dosimeter (Beijing Nuclear Instrument Factory, type FJ427A1). Before collecting data, the powder sample each with weight approximate 0.2 g was pressed into a diameter 10 mm pellet and then the pellet was excited with 365 nm ultraviolet for 30 min to ensure the storage energy of traps have been saturated. The heating rate of TSL measurements was kept at 10 °C/ min. The phases of the phosphors were analyzed with X-ray diffraction (XRD), using the Rigaku D/max-IIIA diffractometer with Cu Ka radiation, operated at 45 kV and 40 mA. The O K-edge and Gd L3-edge X-ray absorption spectroscopy (XAS) was measured on the beamline 20A and 01C1, and the valence band spectra were collected at the beamline 24A of National Synchrotron Radiation Research Centre (NSRRC) in Hsinchu, Taiwan. The electronic band structure (BS), density of states (DOS), partial density of states (PDOS), and charge deformation density (CDD) were calculated, based on density functional theory (DFT). Before calculation, the geometry of the crystal structure was optimized with the generalized gradient approximation (GGA).

## Results and Discussion

### Theoretical calculations on band structure and density of states

**Figure 1 f1:**
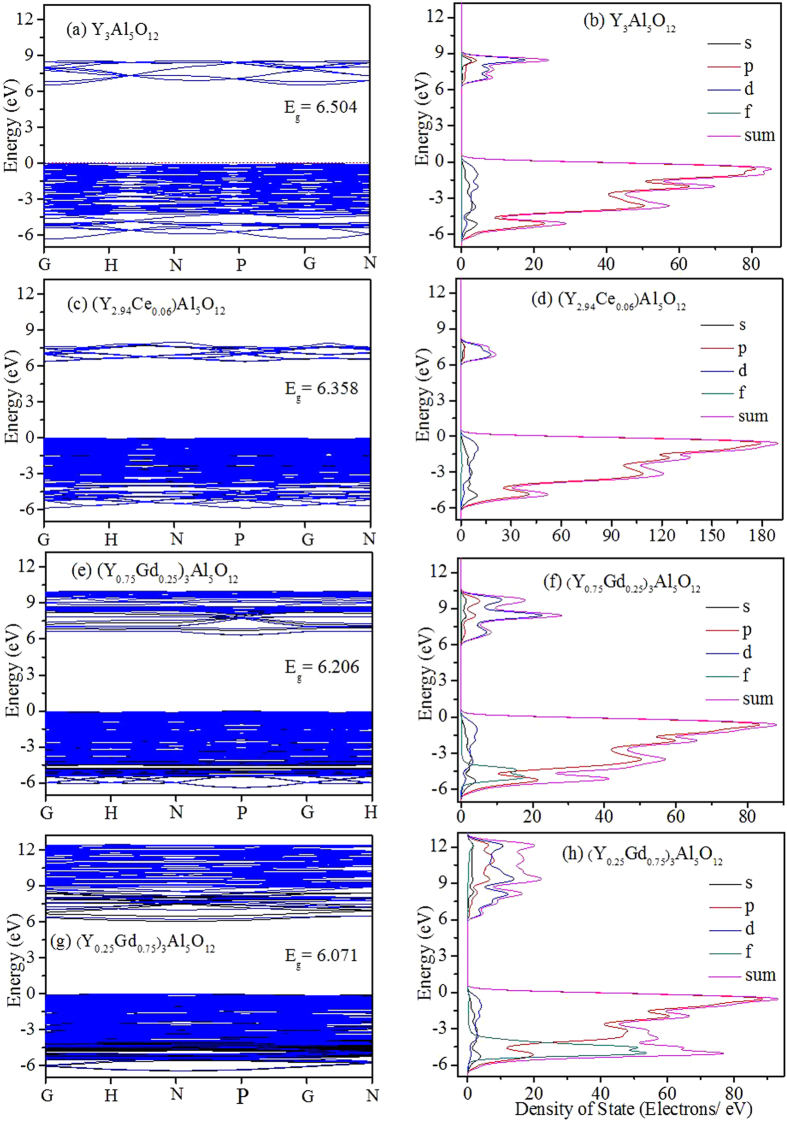
Band structures, density of state, and partial density of state of Y_3_Al_5_O_12_, (Y_2.94_Ce_0.06_)Al_5_O_12_, (Y_0.75_Gd_0.25_)_3_Al_5_O_12_, and (Y_0.25_Gd_0.75_)_3_Al_5_O_12_.

To gain insight into the intrinsic mechanisms of YAG: Ce luminescence upon substitution of Y^3+^ by Gd^3+^, the electronic band structure and density of states were firstly investigated by way of theoretical calculation. Four typical BS, DOS, and PDOS of the pure YAG host and YAG doped with Ce^3+^ and a variant amount of Gd^3+^ are presented in Fig. 1, which shows that the band gap of YAG becomes narrow after doping with Ce^3+^ and Gd^3+^. The calculated band gap of the pure YAG host is 6.505 eV, which is consistent with the value of approximately 6.5 eV evaluated by the photoconductivity method^28^ and the absorption peak at 188 nm determined using spectra method^29^; and the band gaps of (Y_2.94_Ce_0.06_)Al_5_O_12_, (Y_0.75_Gd_0.25_)_3_Al_5_O_12_, and (Y_0.25_Gd_0.75_)_3_Al_5_O_12_ are approximately 6.358, 6.206, and 6.071 eV, respectively. The PDOS in Figs 1(b,d) shows that the top edge of the valence band mainly consists of the O 2p orbital, and the bottom edge of the conduction band mainly consists of the 3d orbital of Y. However, Figs 1(f,h) indicate that the O 2p orbital makes a great contribution to form the conduction band, and the d orbitals (including Y 4d and Gd 5d orbitals) contribute significantly to the valence band after doping with Gd^3+^. This conclusion suggests that the d orbital may hybridize with O 2p orbit intensively after doping with Gd^3+^, further confirmed as follows. The 4f orbital also contributes significantly to the valence band in Figs 1(f,h), but the 4f lies in a deep energy level. In contrast to Figs 1(a,e and g) show that the state density in the conduction band increases intensively as Y^3+^ is replaced with more and more Gd^3+^; moreover, the bandwidth of the valence band enlarges significantly with increasing Gd^3+^. The energy scales of electron spread in the conduction band are approximately 6.5–8.6, 6.2–10.0, and 6.0–12.42 eV, respectively, for x = 0, 0.25, and 0.75 of (Y_1−x_Gd_x_)_3_Al_5_O_12_. The expansion of the conduction bandwidth suggests the extensibility of the atomic orbitals, which consist of the band broadening, the reduction of effective mass of the electron, and crystal field intensification. Meanwhile, the electrons will have a larger non-localization. The calculations on BS, DOS, and PDOS come to the following conclusions: (1) crystal field strength increases, (2) band gap narrows, (3) the effective mass of the electron reduces, and (4) the non-localization expands, as consequences of Gd^3+^ doping. These effects will inevitably affect photoluminescence.

### Photoluminescence properties of (Y_1−x_Gd_x_)_3_Al_5_O_12_: Ce^3+^

**Figure 2 f2:**
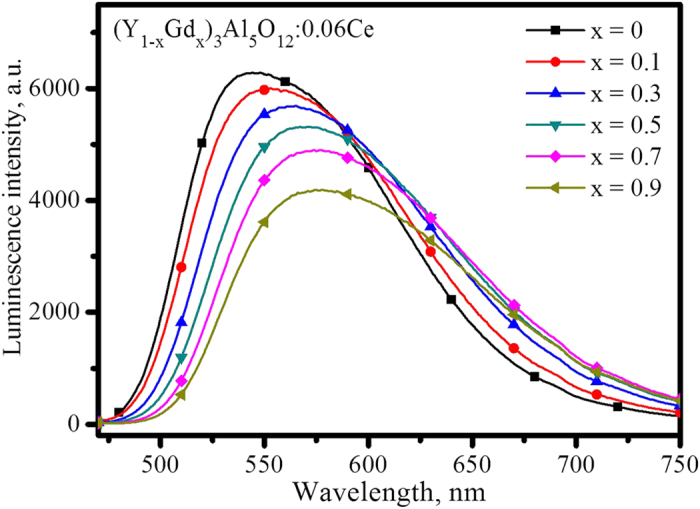
Emission spectra of (Y_1−x_Gd_x_)_3_Al_5_O_12_ excited with 460 nm at room temperature.

**Figure 3 f3:**
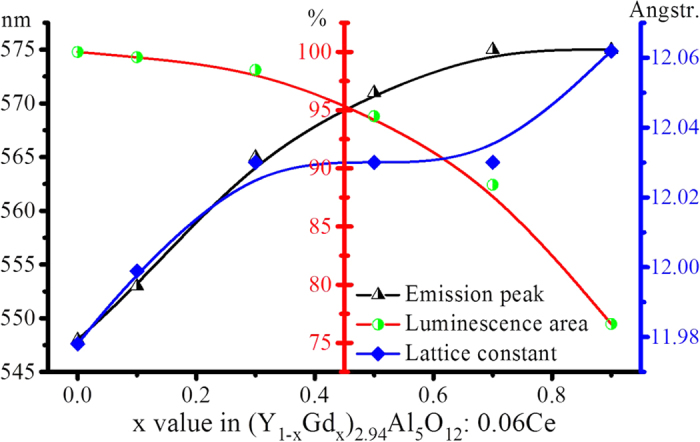
The relationship of emission peak, integrated intensity of luminescence and crystal lattice constant of (Y_1−x_Gd_x_)_3_Al_5_O_12_: Ce as a function of the x value.

Figure 2 presents the emission spectra of (Y_1−x_Gd_x_)_2.94_Al_5_O_12_: 0.06Ce^3+^ (x = 0, 0.1, 0.3, 0.5, 0.7, and 0.9) under the excitation of 460 nm at room temperature, which shows that the luminescence intensity decreases and the emission peak red shifts along with increasing Gd^3+^. As the x value increases from 0 to 0.9, the peak of the emission band shifts from approximately 543 to 575 nm, but the shift is negligible as x changes from 0.5 to 0.9, which can be discriminated more clearly by combining with the normalized emission spectra displayed in supplementary Fig.1. The intensity of luminescence decreases continuously with increasing Gd^3+^, but it decreases rapidly as x varies from 0.5 to 0.9. A quantitative assay shows that the luminescence intensity, achieved by integrating from 480 to 750 nm in emission spectra, decreases approximately 5.5% and 23.4% with x ranges from 0 to 0.5 and 0 to 0.9 (as the red line presented below in Fig. 3 indicates), respectively.

**Figure 4 f4:**
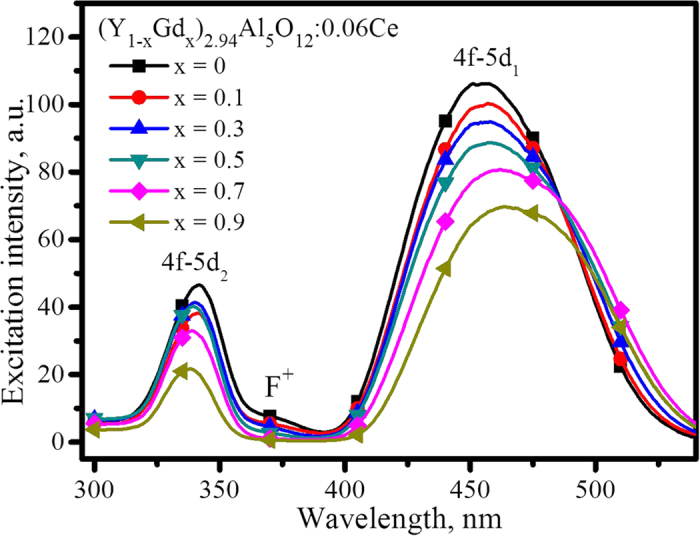
Excitation spectra of (Y_1−x_Gd_x_)_3_Al_5_O_12_ upon the strongest emission at room temperature.

The excitation spectra of (Y_1−x_Gd_x_)_3_Al_5_O_12_: Ce, obtained by monitoring the strongest emission, are shown in Fig. 4. In addition to a minor neighbourhood maximum at 372 nm, two main excitation band peaks at approximately 460 and 340 nm are observed. The Ce^3+^ has [Xe]4f^1^ electronic configuration, the excitation and emission of which originates from the 4f^1^ to 5d^1^ transition. Because of the shielding by the outer 5s and 5p orbitals, the 4f electron is insensitive to the crystal field. However, the 5d electron interacts strongly with the crystal lattice, resulting in strong phonon coupling and large crystal field effects on the excited states. In the crystal lattice of Y_3_Al_5_O_12_, the Ce^3+^ which substitutes for Y^3+^ in the site of D_2_ symmetry has 8 nearest neighbour oxygen atoms. The eightfold coordination of Ce^3+^ in the dodecahedral site can be described as a cubic coordination with an additional tetragonal distortion. According to the ligand field theory, the 5d state will split into a lower energy 5d_e_-doublet state (^2^E_g_) and a higher 5d_t_-triplet state (^2^T_g_) in tetragonal field^25^. All five possible 5d-state energies of Ce^3+^ should be observed in the excitation spectrum, but all five levels were never unambiguously assigned^29^,^30^. Tanner *et al.* confirmed the existence of the two excitation bands located at 342 and 467 nm using the room-temperature Xe-lamp excitation spectra and further revealed another band of Ce^3+^ at 225 nm as well as the YAG host band at 188 nm using the synchrotron radiation excitation spectra^29^. Thus, the two excitation bands at 460 and 340 nm in Fig. 4 could be attributed to the 4f–5d_1_ and 4f–5d_2_ transitions, respectively.

The 4f-5d transition of Ce^3+^ emission with asymmetric broadband configuration in Fig. 2 consists of doublet sub-emissions from 5d_1_ to ^2^F_2/7_ and 5d_1_ to ^2^F_5/2_, because the ground state of Ce^3+^ consists of ^2^F_2/7_ and ^2^F_5/2_ sublevels after considering the spin-orbit interaction^19^,^29^. The minor band with a peak at approximately 372 nm (26880 cm^−1^) in Fig. 4 was reported in the literatures^21^,^23^,^24^,^25^,^31^,^32^,^33^,^34^,^35^,^36^,^37^,^38^,^39^,^40^ but was not apparent in other studies^16^,^18^,^19^,^20^,^25^,^28^,^29^,^30^. Gracia calculated the absorption and luminescence spectra of Ce^3+^ doped YAG using an ab initio embedded cluster approach and concluded that a small peak at 372 nm is not due to Ce^3+^ ions^41^. Zeng attributed this band to an F-type colour centre^39^. Through studying a YAG: Ce single crystal, grown with temperature gradient techniques (TGT), by means of thermal annealing in a H_2_ and O_2_ atmosphere combining with different doses of gamma irradiation, Dong further demonstrated that it was a type of F^+^-type colour centre^32^.

Both the intensity of 4f-5d_1_ excitation at 460 nm and the excitation of 4f-5d_2_ at 340 nm in Fig. 4 decrease with an increase of the x value, which is helpful to illustrate the luminescence decrease in Fig. 2. After normalizing the strong excitation to 1.0, however, the supplementary Fig. 2 shows that the relative excitation intensity of the band at 340 nm increases as x increases from 0 to 0.5 and then decreases as x increases onward to 0.9. This phenomenon puts forward an interesting topic regarding the relative distribution of electrons on different energy levels; this population usually obeys the Fermi-Dirac distribution function (seen below Equation (6)), depending on the energy barrier and temperature.

**Figure 5 f5:**
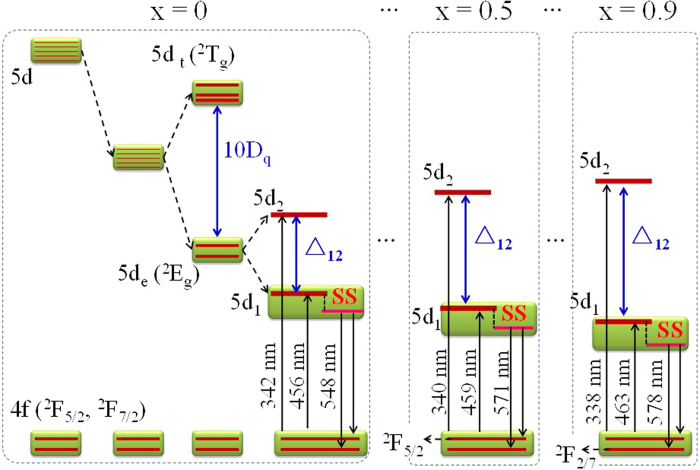
The crystal field splitting and the energy level diagram for Ce^3+^ in (Y_1−x_Gd_x_)_3_Al_5_O_12_ upon substitution of Y^3+^ by Gd^3+^ and its excitation and emission processes, where Δ = 10D_q_ indicates the crystal splitting energy acting on the 5d_e_-doublet state (^2^E_g_) and the 5d_t_-triplet state (^2^T_g_) in tetragonal field, Δ_12_ indicates an additional crystal splitting energy on the 5d_e_-doublet state to form the 5d_1_ and 5d_2_ states, the SS denotes Stokes shift. The splitting and transition involving the 5d_t_ states are omitted.

As x increases from 0 to 0.9, the band of 4f-5d_1_ excitation shifts towards longer wavelengths whereas that of 4f-5d_2_ shifts towards shorter wavelengths, as shown in Fig. 4. This phenomenon can be seen more clearly in supplementary Fig. 2. The shift in opposite directions of two excitation bands indicates the splitting of the Ce^3+^ 5d state enlarges with increasing Gd^3+^, caused by the intensified crystal field. This conclusion is consistent with the above prediction of the theoretical calculation. Thus, the diagram of Ce^3+^ energy levels and the crystal-field splitting of 5d orbitals in (Y_1−x_Gd_x_)_3_Al_5_O_12_ could be described with Fig. 5. With more and more Y^3+^ replaced by Gd^3+^, both the crystal-field splitting and Stokes shift increase with an increase of the x value, as seen from the quantitative values summarized in Table 1.

Additionally, Fig. 4 shows that the intensity of the band with a peak at 372 nm decreases step-by-step when with more and more Gd^3+^ is doped into the YAG, suggesting the doped Gd^3+^ is helpful in removing the F^+^ centres.

### Diffuse reflection and absorption of (Y_1−x_Gd_x_)_3_Al_5_O_12_: Ce^3+^

**Figure 6 f6:**
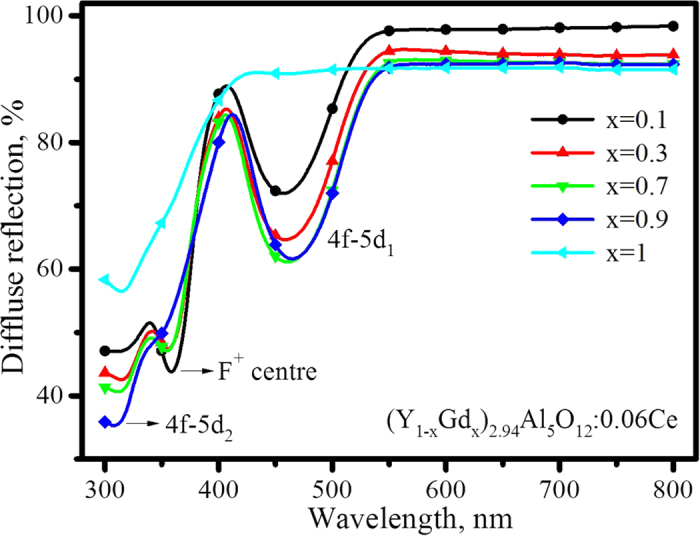
Diffuse reflection spectra of (Y_1−x_Gd_x_)_2.94_Al_5_O_12_: 0.06Ce^3+^.

The intensity of excitation reflects the comprehensive effects of energy absorption and energy transfer in the phosphor. To confirm that the decrease of (Y_1−x_Gd_x_)_3_Al_5_O_12_: Ce^3+^ luminescence upon Gd^3+^ doping was not caused by the reduced absorption, the diffuse reflection spectra were measured. As presented in Fig. 6, the two main absorption bands observed in the regions of 410–550 nm and 330–370 nm, respectively, correspond to the two excitation bands of 4f–5d_1_ and 4f–5d_2_, previously shown in Fig. 4 as well. The spectral shifts of the 4f–5d_1_ and 4f-5d_2_ absorption bands in two opposite directions were also observed. Differing from the excitation spectra displayed in Fig. 4, both the absorption intensity of 4f–5d_1_ and the absorption of 4f–5d_2_ increase as Gd^3+^ concentration increases from x = 0.1 to 0.9. In addition, Fig. 6 shows that the relative diffuse reflection intensity (including background) decreases as Gd^3+^ increases from x = 0.1 to 0.9 in the full wavelength range of 300 to 800 nm, except for the absorption of F^+^ centres. With the background intensity of diffuse reflection normalized to 1.0, as shown in supplementary Fig. 3, the conclusion that absorption intensity increases with Gd^3+^ holds true. Therefore, the decrease of (Y_1−x_Gd_x_)_3_Al_5_O_12_: Ce^3+^ luminescence upon doping with Gd^3+^ is not caused by the reduced absorption.

It must be noted that the excitation intensity of 4f-5d_1_ is far stronger than that of 4f-5d_2_ in Fig. 4; however, the relative absorption intensity of 4f-5d_2_ is much stronger than that of 4f-5d_1_ in Fig. 6. The difference between the absorption area in Fig. 6 and the excitation area in Fig. 4 suggests most of the absorption energy of 4f-5d_2_ is lost without emission. This phenomenon indicates that the transition of 4f-5d_2_ has a strong ability to absorb incident photons, but only a fraction of them convert to visible light. The energy loss of the 5d_2_ state may be caused by the transition from 5d_2_ to the conduction band, as discussed below.

**Figure 7:**
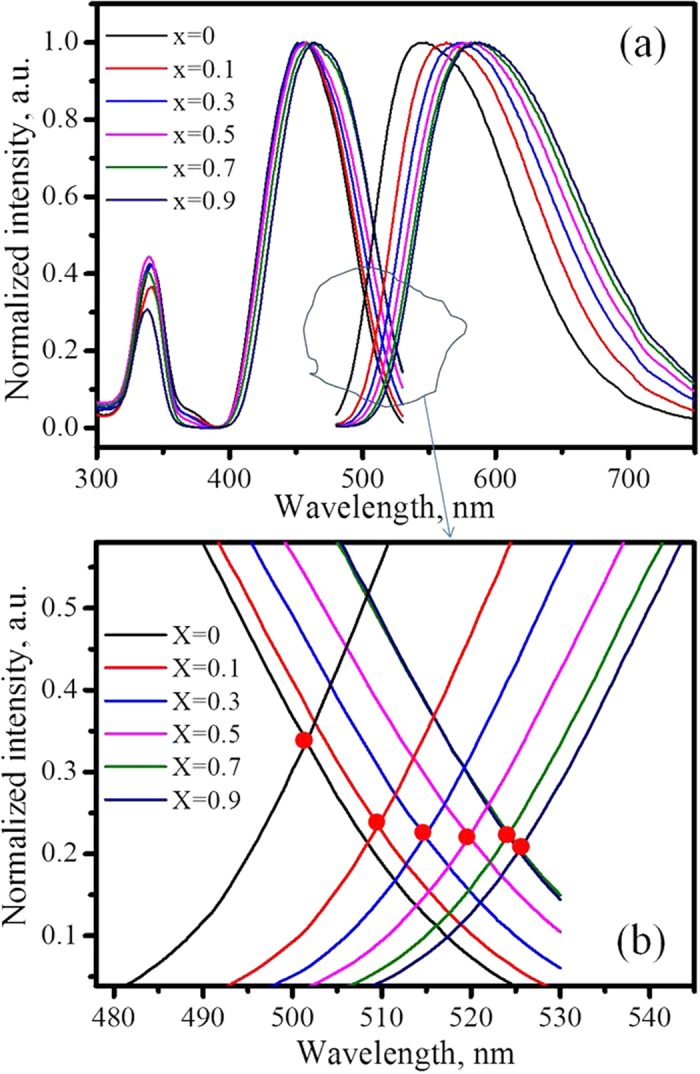
The emission and excitation spectra of (Y_1−x_Gd_x_)_2.94_Al_5_O_12_: 0.06Ce^3+^ with normalized intensity (a) and the amplified region showing the crossover of the emission and excitation upon different amount of Gd^3+^.

Figure 7(a) plots the normalized emission and excitation spectra of (Y_1−x_Gd_x_)_2.94_Al_5_O_12_: 0.06Ce^3+^. Partial enlarged detail showing the crossover of the emission and excitation spectra with different amounts of Gd^3+^ is presented in Fig. 7(b), which shows that the crossover shifts towards longer wavelengths as x value increases from 0 to 0.9. According to the coordination-configuration theory, the energy loss of the non-radiative transition from the excited state to the ground state is proportional to the integral of the emission and excitation spectra, i.e.,


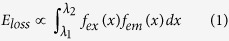


where the f_ex_(x) and f_em_(x) denote the wave functions of the emission and excitation spectra, respectively. However, the overlapping area below the curves of the emission and excitation spectra for each sample decreases with an increase of the x value. Therefore, the nonradiative transition of electrons relaxed from the 5d_1_ excited state through the crossover of the potential parabola curves to the 4f ground state is not the main mechanism of energy loss.

### Thermal stability luminescence of (Y_1−x_Gd_x_)_3_Al_5_O_12_: Ce^3+^

**Figure 8:**
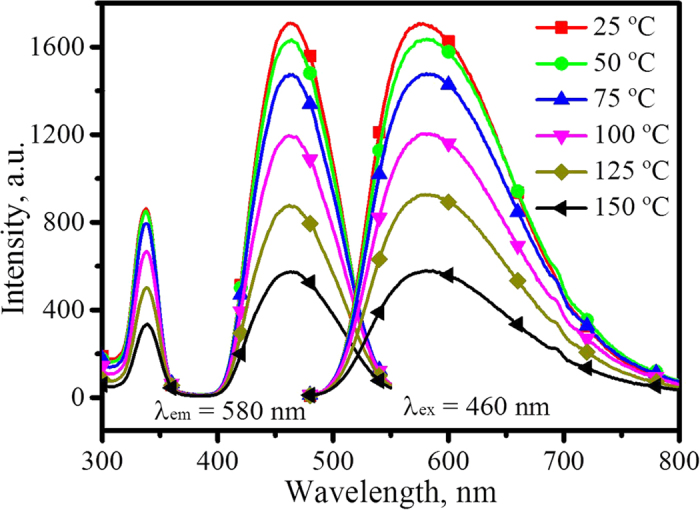
Emission and excitation spectra of (Y_0.1_Gd_0.9_)_2.94_Al_5_O_12_: 0.06Ce^3+^ at various temperatures.

Chromaticity shift and luminescence quenching upon temperature changes are unfavourable for a phosphor applied in white LEDs, thus the thermal stability of luminescence should be carefully examined. The emission and excitation spectra measured at various temperatures of one typical sample with the maximum x = 0.9 for (Y_1−x_Gd_x_)_3_Al_5_O_12_: Ce^3+^ is provided in Fig. 8, which shows that excitation and emission intensity decrease rapidly with an increase of temperature. The relative luminescence intensities, achieved by integrating from 480 to 750 nm and with the intensity of each sample luminescence at room temperature normalized to 100%, of samples with x = 0, 0.3, 0.5. 0.7, and 0.9 for (Y_1−x_Gd_x_)_3_Al_5_O_12_: Ce^3+^ as function of temperature are presented in supplementary Fig. 4. This shows that the relative intensity decreases more and more along with increasing Gd^3+^. As the temperature increases from room temperature to 125 °C, the relative intensity of the YAG: Ce decreases approximately 4%, but (Y_0.1_Gd_0.9_)_2.94_Al_5_O_12_: 0.06Ce^3+^ luminescence decreases approximately 64%. According to configuration coordinate theory, a heavy weight should benefit in resisting thermal vibration and reducing phonons, and accordingly the thermal stability of YAG: Ce luminescence should enhance with Gd^3+^ doping. An evident spectral shift of YAG: Ce emission upon a change in temperature has been observed with and without a small amount of Gd^3+^ doping, as seen from Fig. 5 in reference^19^. However, with a large amount of Gd^3+^ doping, as in this work, no spectral shift upon temperature change is observed in the emission spectra of (Y_0.1_Gd_0.9_)_2.94_Al_5_O_12_: 0.06Ce^3+^, as presented in Fig. 8, and the spectra with normalized intensity are displayed in supplementary Fig. 5.

In configuration coordinate theory, the spectrum either red shifts (due to coupling with phonons) or blue shifts (when transiting from a low energy level to a high one by coupling with phonons to give light). Here, no spectral shift but a serious decrease of luminescence occurs, which should be caused by the ionization of electrons from excited states to the conduction band (as discussed below). Moreover, the non-shift of emission suggests that doping Gd^3+^ into YAG: Ce makes the structure more rigid than it was previously. Both the luminescence decreases and the spectral non-shift behaviours upon temperature change are out of the range that the configuration coordinate diagram model can explain. The red shift of (Y_1−x_Gd_x_)_3_Al_5_O_12_: Ce^3+^ emission as function of Gd^3+^ concentration at room temperature could be interpreted according to the intensified crystal field, but what mechanism has resulted in the increase of crystal field strength deserves more study. To explore these mysteries, the electronic and crystal structures were examined.

### Electronic structure and the mechanism of spectra shift

**Figure 9:**
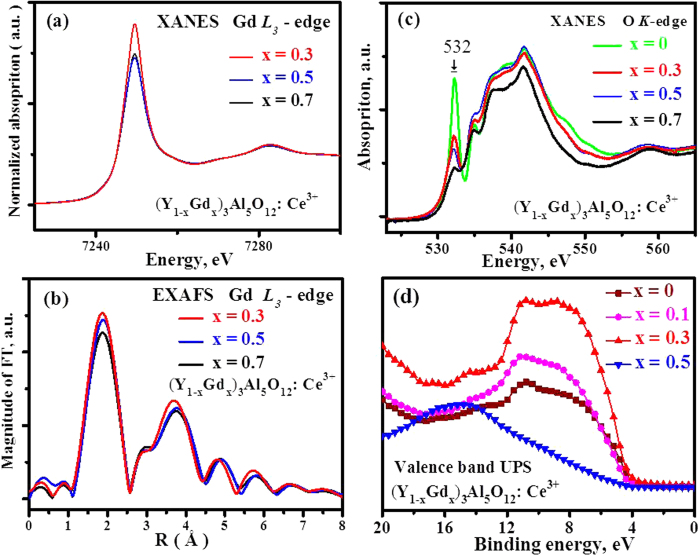
(**a**) The Gd L_3_-edge XANES, (**b**) the Fourier transformation EXAFS spectra in R space, (**c**) the O K-edge XANES spectra, and (**d**) the valence band spectra of (Y_1−x_Gd_x_)_3_Al_5_O_12_: Ce^3+^.

It is well known that the XANES spectra reflect the unoccupied density of states restricted by the dipole selection. Thus, XANES spectra could give us information on the geometric and electronic structure of the chemical bonding situation and the effective charge density around the X-ray-absorbing atoms. Because the low concentration of Ce^3+^ was out of the sensitivity of the instrument, the XANES spectra monitoring the Gd^3+^ L_3_-edge were preferentially measured. The sharp lines presented in Fig. 9(a) were caused by the electron transition from Gd 2p_3/2_ to outer unoccupied 5d orbitals, whose absorption intensity decreases with an increase of x value from 0.3 to 0.7 for (Y_1−x_Gd_x_)_3_Al_5_O_12_: Ce^3+^. This point is consistent with the decrease of the main peak of the EXAFS spectra in the Fourier transformation R space in Fig. 9(b). The absorption decrease in intensity along with increasing Gd^3+^ indicates that the outer unoccupied 5d orbital is filled with more and more electrons. Figure 9(c) presents the O K-edge XANES spectra of (Y_1−x_Gd_x_)_3_Al_5_O_12_: Ce^3+^ (x = 0, 0.3, 0.5, and 0.7), in which the sharp peak of the absorption at 532 eV is attributed to the excitation of O 1s electrons to O 2p states strongly hybridized with Y 3d or Gd 4f states. The sharp peak of the absorption at 532 eV decreases continuously with an increase of Gd^3+^ concentration from x = 0 to x = 0.7, suggesting that the outer unoccupied 2p orbital of O was also filled with electrons. The presence of sharp line absorption peaks of XANES, called the Rydberg states, indicates the atomic orbitals keep well. If the molecular orbital were formed through hybridization, the Rydberg state would not be sharp, and the broadband configuration would be present. A possible reason for the unoccupied Gd 5d and O 2p simultaneously filled with electrons is through orbital hybridization to a form molecular orbital.

Because Gd^3+^ has smaller electronegativity than Y^3+^, Gd^3+^ should have a poorer ability than Y^3+^ to hybridize 5d with O 2p in theory, but it does not practically. Ram’s ^[15]^ conclusion about the strong rigidity of the YAG structure with small atomic displacement parameters provides us with a useful clue to understanding this phenomenon. Because of the larger radius, Gd^3+^ ions will suffer a compressive resistance from neighbouring atoms when incorporated into the rigid crystal lattice of YAG. In the YAG crystal lattice, Gd^3+^ takes the place of the Y^3+^ site with high symmetry for its eightfold coordination. Moreover, the Gd 5d orbital could spread in a large space in contrast to the Y 4d orbital due to its larger radius. Under the comprehensive effect of above factors, the strong compression effect in a high symmetric site compels the Gd 5d orbital to hybridize with the O 2p orbital.

To support this viewpoint, the existence of strong compression was demonstrated firstly. As for a cubic garnet structure, the spacing d between adjacent (hkl) lattice planes obeys the relationship:


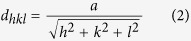


The XRD patterns of (Y_1−x_Gd_x_)_3_Al_5_O_12_: Ce^3+^ (x = 0, 0.1, 0.3, 0.5, 0.7, 0.9, and 1.0) are presented in supplementary Fig. 6, in which all diffraction peaks can be indexed to the standard Y_3_Al_5_O_12_ (JCPDS: 33-0040) when the value of x is no higher than 0.3. However, the minor phase of GdAlO_3_ (JCPDS: 46-0395) is indexed as x = 0.3 to 0.9. The 2θ value of the strongest diffraction peak at the crystal planes (420) decreases with increasing Gd^3+^ from x = 0 to 0.9, as shown on the right side in supplementary Fig. 6, with an amplified range of 33–34°, indicating an increase of the crystal lattice parameter. A linear expansion of the lattice constant as function of Gd^3+^ concentration is found within x ≤ 0.3 in Fig. 3, but it increases nonlinearly when x > 0.3. Such a nonlinear curve is below the line determined with the same slope as x ≤ 0.3, indicating the measured crystal lattice constants of samples doped with Gd^3+^ are lower than the theoretical value predicted with Vegard’s law.

However, the EXAFS data could provide information about the inter-atomic distances and the coordination. The eightfold coordination has been confirmed. Figure 9(b) shows that the first neighbour shell distances in the R space of Fourier transformation EXAFS spectra of (Y_1−x_Gd_x_)_3_Al_5_O_12_: Ce^3+^ are approximately 1.8715, 1.9021, and 1.8715 Å for x = 0.3, 0.5 and 0.7, respectively. The deviations of the R values are within experimental error. Therefore, the measured data reveal that there is virtually no increase in bond length with Gd^3+^ doping in the concentration range from x = 0.3 to 0.7. However, the radius of Gd^3+^ is larger than Y^3+^. The result indicates that the crystal lattice after Gd^3+^ doping is smaller than the ideal volume. The shrink of the crystal lattice volume suggests that the doped Gd^3+^ ions receive an intensive squeezing effect from other atoms. When more and more Gd^3+^ is introduced into the lattice, the compressive stress will increase exponentially. Accordingly, the emission wavelength increases nonlinearly, as shown in Fig. 3, as a consequence of the intensified crystal field. The two curves, i.e., luminescence intensity and emission wavelength as function of Gd^3+^ concentrations, are nearly axisymmetric along the vertical axis. The profile of these two curves looks like a butterfly, which can be called the butterfly effect for the compression simultaneously activated on luminescence intensity and emission wavelength.

**Figure 10:**
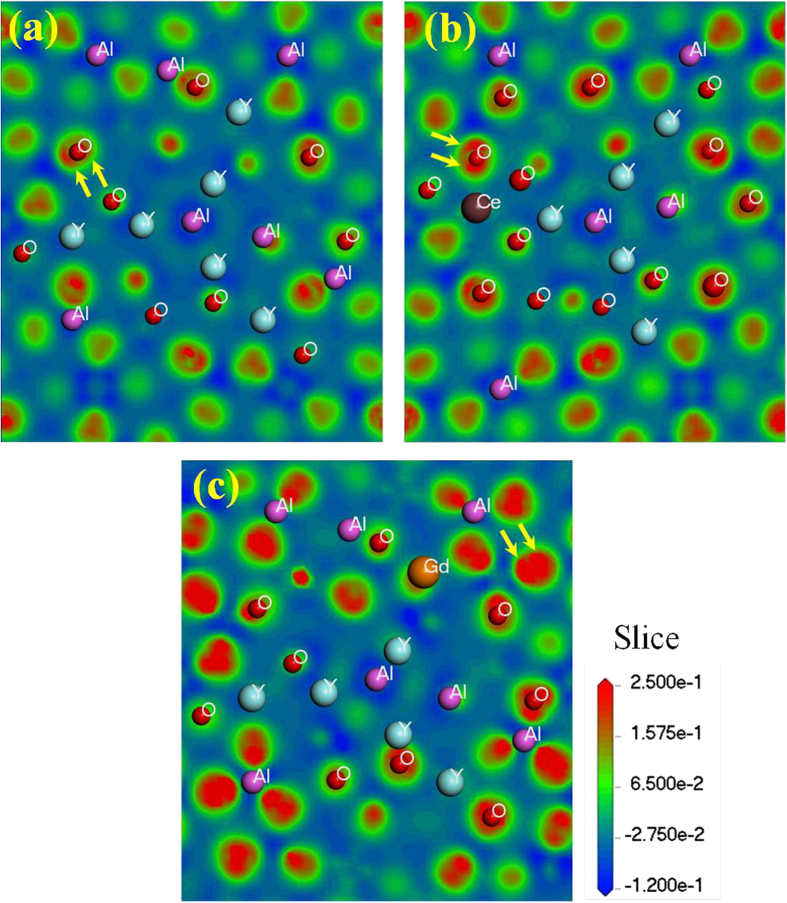
Charge deformation density of Y_3_Al_5_O_12_ (**a**), (Y_0.98_Ce_0.02_)_3_Al_5_O_12_ (**b**), and (Y_0.75_Gd_0.25_)_3_Al_5_O_12_ (**c**) calculated based on DFT.

Finally, a theoretical calculation on CDD provides visual evidence regarding the change of charge distribution after doping Ce^3+^ and Gd^3+^ into YAG. When there is no Ce^3+^ or Gd^3+^ doped in the pure YAG host, as presented in Fig. 10(a), the charge is restricted to a certain space. The blue colour around the Al atoms indicate that they lose electrons, and the red colour around the O atoms indicate that they gain electrons. With respect to Fig. 10(a), the intensified red colour around the O atoms in Figs 10(b,c) shows that the charge density around oxygen increases, either doping Ce^3+^ or doping Gd^3+^ into the YAG. Especially for Gd^3+^ doping, the charge density around oxygen increases significantly. Moreover, the dumbbell shape of the p orbitals around O could be discriminated in Figs 10(a,b), as the arrows indicated; however, in Fig. 10(c) they combine into one ellipsoid under the compression effect, which provides a visual picture of the deformation of the electron cloud. Not only does the charge density around oxygen intensify but also the volume of electron cloud expands after doping with Gd^3+^ and Ce^3+^, due to the hybrid orbital. As far as the charge density around Y^3+^ is concerned, the comparison of Figs 10(a,c) shows that the charge density around Y^3+^ increases a little after Gd^3+^ doping. These characteristics indicate that the orbital hybridization between Y and O intensifies with Gd^3+^ doping, consistent with the result displayed in Figs 8(a,b). The electronic structure changes in turn, which can explain the BS and DOS picture presented in Fig. 1. At least, the variation of the valence band structure is observed in this experiment, as shown in Fig. 9(d).

Compared with the space of electron spread in Fig. 10(a), an obvious expansion of the electron cloud around oxygen is observed in Fig. 10(b), which makes the effective Ce-O bond length shorten. According to the relationship between crystal field strength and ionic bond length:


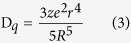


where the parameter D_q_ presents the crystal field stabilization energy (CFSE), R is the bond length between a central ion and ligand ions, r is the mean size of the central ion, and Z is the charge of a central ion. Thus, the shorter bond length implies the stronger crystal field strength. Accordingly, the 5d state will have a larger split after doping with Gd^3+^, which can explain the results in Table 1.

The change of the electron cloud can explain the variation of band structure and the density of states of YAG upon doping with Ce^3+^ and Gd^3+^ as well. Because of the orbital hybridization, electrons can spread in a large space. With the expansion of the electron cloud around O atoms, the band gap of (Y_1−x_Gd_x_)_3_Al_5_O_12_ becomes narrow and the density of states becomes dense. The intensified crystal field will reduce the effective mass of the electrons and make them move easily, which will decrease the thermal stability of luminescence. In addition, the expansion of the electron cloud around oxygen makes it easy for electrons to be promoted to excited states. The electronic and crystal structures obtained here provide insight into the photoluminescence properties.

### Crystal defects and trap depths

**Figure 11:**
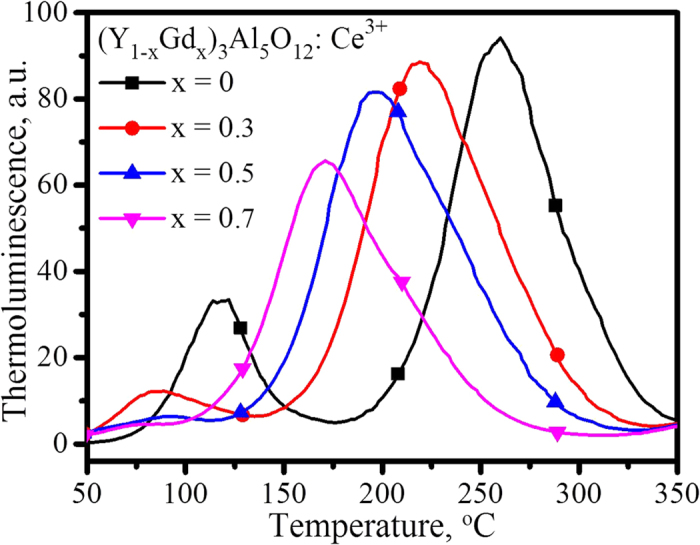
Thermally stimulated luminescence of (Y_1−x_Gd_x_)_2.94_Al_5_O_12_: 0.06Ce^3+^.

**Figure 12:**
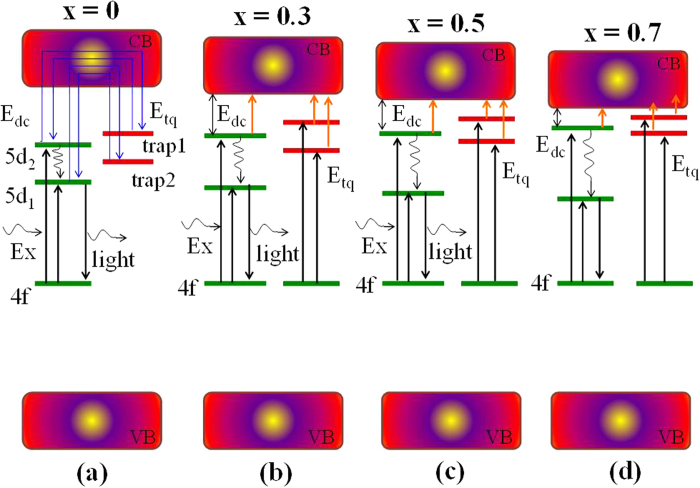
The relative position of 5d_1_ and 5d_2_ of Ce^3+^ and the relative depth of trap 1 and trap 2 in the band structure of (Y_1−x_Gd_x_)_3_Al_5_O_12_: Ce^3+^.

However, the performance of a phosphor, including luminescence efficiency and thermal stability, is closed related to crystal defects. The thermoluminescence which was considered the best tool in identifying crystal defects was used to characterize the YAG: Ce phosphor. Figure 11 shows the TSL of (Y_1−x_Gd_x_)_2.94_Al_5_O_12_: 0.06Ce^3+^, in which two emission bands are observed: a weak band in the range of 50–150 °C and a strong one in 125–350 °C. The peak of the weak band shifts from approximately 118 °C to 85 °C as x value increases from 0 to 0.3, accompanied by a decrease in intensity; and then the peak almost disappears as x value increases onwards to 0.5 and 0.7. However, the peak of the strong band shifts continuously with an increase of Gd^3+^ concentration, from approximately 260 °C at x = 0, via 219 °C at x = 0.3 and 197 °C at x = 0.5, and finally reaches 171 °C at x = 0.7, in addition to a continuous decrease of luminescence intensity. The thermally stimulated luminescence shows that the following processes happened: when electrons are excited to the high-energy state, some of them are captured by the defects, and then they are thermally released from the defects to the excited state, finally giving light during the transition from a high-level excited state to the ground state, as the blue line depicted in Fig. 12. Previously reports on the persistent luminescence of YAG: Ce also confirms the existence of crystal defects^42^,^43^. The decrease in the intensity of TSL suggests the decrease of the concentration of crystal defects; and the shift of TLS peak from high to low temperature indicates the decrease of the trap depth. Two TSL peaks indicate the existence of two types of defects in the phosphor. The order of kinetics of the glow curves was determined with the peak shape method, by calculating the symmetry (geometrical) factor:


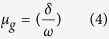


where ω = Τ_2_−Τ_1_ denotes the full width of the glow peak at half its maximum height, τ = T_m_−T_1_ presents the low-temperature half width, and δ = T_2_ − Τ_m_ is the high-temperature half width. The values of the geometrical factor suggest that the peaks obey general order kinetics. Thus, the trap depth could be calculated using Chen’s equation^44^:


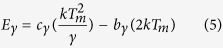


where k is the Boltzmann constant and T_m_ is the peak temperature. The calculated data are shown in Table 2. YAG is a high-dielectric material, whose permittivity is approximately 11.7. The high permittivity indicates that a strong polarity (or dipoles) exists in the local circumstance of YAG, which will cause (Y,Gd)_3_Al_5_O_12_: Ce^3+^ to have a strong surface adsorption, such as the OH stretching bands observed in the infrared absorption spectra of YAG^32^,^33^,^34^,^35^,^36^,^37^. The surface adsorption will change the surface energy levels and finally affect the luminescence. Thus, we are inclined to attribute the emission in the range of 50–150 °C to the defect of surface adsorption and the other in 125–350 °C to the F^+^ centres caused by O_h_^-^ oxygen vacancies^32^,^33^,^34^,^35^.

### Mechanisms of luminescence decrease and thermal quenching of YAG: Ce upon doping with Gd^3+^

The population of electrons in different energy levels obeys the Fermi-Dirac distribution:


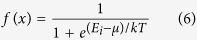


where T is the absolute temperature, k is Boltzmann’s constant, E_i_ is the energy of the single-particle state i, and μ is the total chemical potential. With more and more Gd^3+^ doped into the YAG, one side the band gap of (Y_1−x_Gd_x_)_3_Al_5_O_12_: Ce^3+^ decreases; on the other side, the energy splitting between 5d_1_ and 5d_2_ expands, which makes the 5d_2_ energy level of Ce^3+^ approach the bottom of the conduction band. When the electrons are excited from 4f to 5d_1_ state to produce blue light for white LED applications, assuredly certain amount of electrons will be excited to the 5d_2_ state according to the Fermi-Dirac distribution. Moreover, the population of electrons in the 5d_2_ state will increase with increasing Gd^3+^, due to the reduced energy barrier between 5d_2_ and the conduction band. This mechanism can explain the phenomenon of the 4f-5d_2_ absorption of Ce^3+^ enhanced evidently in Fig. 6, but the excitation in Fig. 4 does not rise correspondingly upon doping with Gd^3+^. Under the effect of temperature, the electrons are easily ionized from 5d_2_ to the conduction band to produce photocurrent. Moreover, the electrons are possibly captured by crystal defects.

The increase of Gd^3+^ concentration reduces the trap depth of crystal defects, which results in the weakly trapped electrons being easily released to the conduction band. Once the excited electrons are captured by crystal defects, they possibly delocalize to the conduction band with the help of temperature. The mechanism of thermal delocalization of electrons from crystal defects to the conduction band can explain the serious thermal luminescence quenching that occurs in Fig. 8, although a spectral shift with increasing temperature is not observed. Therefore, the auto-ionization of electrons from 5d_2_ to the conduction band and the thermal delocalization of electrons from crystal defects to the conduction band are the main mechanisms of energy loss involved in (Y_1−x_Gd_x_)_3_Al_5_O_12_: Ce^3+^ luminescence. Although the band gaps decrease with increasing Gd^3+^, Fig. 9(d) shows that the position of the valence band relative to Fermi level does not significantly change. Thus, the processes of energy loss and luminescence could be described in Fig. 12.

## Conclusion

In summary, a new interpretation from the viewpoint of compression deformation of the electron cloud in a rigid structure by combining hybrid orbital theory with solid-state energy band theory together has been put forward in this work to illustrate the intrinsic mechanisms that cause the emission spectral shift, thermal quenching, and decrease in intensity of YAG: Ce luminescence upon substitution of Y^3+^ by Gd^3+^. Due to Gd^3+^ having a larger radius in comparison with Y^3+^, Gd^3+^ is subject to a strongly compressive effect when it takes the place of the eightfold coordinated Y site in the rigid YAG crystal lattice, which compels the Gd 5d and Y 4d orbitals to hybridize with the O 2p orbital to form a molecular orbital. Because of the orbital hybridization, electrons can spread over a large scale of energy levels and the band gap becomes narrow as increasingly more Y^3+^ is replaced by Gd^3+^. The intensified crystal field reduces the effective mass of the electrons, and the sub-state of Ce^3+^ 5d orbital splitting expands. Two types of crystal defects, a shallow trap caused by surface adsorption and another deep F^+^ centre, were identified; and the trap depths decrease with increasing Gd^3+^. In addition to the narrow band gap, the energy barriers for electron transition from the 5d_2_ sublevel and crystal traps to the conduction band reduce with an increase in Gd^3+^ concentration. Accordingly, the electrons easily auto-ionize or de-localize into conduction band. The auto-ionization of electron from 5d_2_ to the conduction band and thermal delocalization of electrons from crystal traps to the conduction band are the main mechanisms of luminescence decrease and thermal quenching of (Y,Gd)AG: Ce upon doping with Gd^3+^. The theoretical calculation of the charge deformation density provides a visualized picture of electron cloud expansion, especially for the expansion of the electron cloud around O atoms which makes the effective Ce-O bond length shorten, further resulting in an increase of crystal field strength and spectral shift. This work provides a solid foundation to tune emission colour in rigid structure, such as in diamond, corundum, and high-strength nitrides, using charge deformation. However, the band gaps and crystal defects must be strictly controlled in band gap engineering, in comprehensive consideration of the luminescence thermal stability and efficiency.

## Additional Information

**How to cite this article**: Chen, L. *et al.* Charge deformation and orbital hybridization: intrinsic mechanisms on tunable chromaticity of Y_3_Al_5_O_12_:Ce^3+^ luminescence by doping Gd^3+^ for warm white LEDs. *Sci. Rep.* **5**, 11514; doi: 10.1038/srep11514 (2015).

## Supplementary Material

Supplementary Information

## Figures and Tables

**Table 1:** The centroid of the 5d_1_ and 5d_2_ excited states, crystal field splitting energy, and the Stokes shift within the ground state of 4f and the lowest excited state of 5d_1_ for Ce^3+^ in (Y_1−x_Gd_x_)_3_Al_5_O_12_.

	Excitation peak of 5d_1_ (nm)	Excitation peak of 5d_2_ (nm)	Crystal field splitting energy Δ_12_ (eV)	Emission peak (nm)	Stokes Shift within 5d_1_ and 4f (nm)
x = 0	342 (3.6257 eV)	456 (2.7193 eV)	0.9064	548	92
x = 0.1	342 (3.6257 eV)	457 (2.7134 eV)	0.9124	553	96
x = 0.3	340 (3.6471 eV)	458 (2.7074 eV)	0.9396	565	107
x = 0.5	340 (3.6471 eV)	459 (2.7015 eV)	0.9455	571	112
x = 0.7	339 (3.6578 eV)	461 (2.6898 eV)	0.9680	577	116
x = 0.9	338 (3.6686 eV)	463 (2.6782 eV)	0.9905	578	115

**Table 2:** The temperature of maxima thermoluminescence, geometrical factor, and trap depth in (Y _1−x_Gd_x_)_2.94_Al_5_O_12_: 0.06Ce^ 3+^.

Gd^3+^ concentration	Defect types	T_m_ (°C)	Geometrical factor	E_τ_	E_δ_	E_ω_	Mean trap depth (eV)
x = 0	Trap 1	117.4305	0.4818	0.67216	0.99124	1.08838	0.91726
	Trap 2	260.0099	0.5296	0.80031	1.37887	1.58086	1.25335
x = 0.3	Trap 1	89.505	0.5169	0.42419	0.75142	0.85958	0.6784
	Trap 2	218.5566	0.5821	0.48628	1.03064	1.28252	0.93315
x = 0.5	Trap1	/	/	/	/	/	/
	Trap 2	205.0944	0.6232	0.39772	0.94081	1.24951	0.86268
x = 0.7	Trap1	/	/	/	/	/	/
	Trap 2	171.147	0.6119	0.3998	0.91307	1.18843	0.83376
