# Supplementary material for: Charge deformation and orbital hybridization: intrinsic mechanisms on tunable chromaticity of Y3Al5O12:Ce3+ luminescence by doping Gd3+ for warm white LEDs
Source: Sci Rep. 2015 Jul 15;5:11514. doi: 10.1038/srep11514 (PMC4502399; doi:10.1038/srep11514)
Supplement: Supplementary Information [file srep11514-s1.doc]

Charge deformation and orbital hybridization: intrinsic mechanisms on tunable chromaticity of Y_3_Al_5_O_12_:Ce^3+^ luminescence by doping Gd^3+^ for warm white LEDs

Lei Chen [[1]](#footnote-2),*, Xiuling Chen ^1^, Fayong Liu ^1^, Haohong Chen ^2^, Hui Wang ^1^, Erlong Zhao ^1^, Yang Jiang ^1,*^, Ting-Shan Chan ^3^, Chia-Hsin Wang ^3^, Wenhua Zhang ^4^, Yu Wang ^5^, Shifu Chen ^6,*^

*^1^ School of Materials Science and Engineering, Hefei University of Technology, Hefei 230009, China.*

*^2^ Shanghai Institute of Ceramics, Chinese Academy of Sciences, Shanghai 200050, China*

*^3^ National Synchrotron Radiation Research Center, HSinchu 30076, Taiwan*

^4^ National synchrotron Radiation Laboratory, University of Science and Technology, Hefei 230026, China

*^5^ Shanghai Synchrotron Radiation Facility, Shanghai 201204, China*

*^6^ Department of Chemistry, Anhui Science and Technology University, Fengyang 233100, China*

SFig. 1 Normalized emission spectra of (Y_1-x_Gd_x_)_3_Al_5_O_12_ excited with 460 nm at room temperature

SFig. 2 Normalized excitation spectra of (Y_1-x_Gd_x_)_2.94_Al_5_O_12_: 0.06Ce^3+^

SFig. 3 Diffuse reflection spectra of (Y_1-x_Gd_x_)_2.94_Al_5_O_12_: 0.06Ce^3+^ with background intensity normalized

SFig. 4 Luminescence intensity of (Y_1-x_Gd_x_)_2.94_Al_5_O_12_: 0.06Ce^3+^ (x = 0, 0.3, 0.5. 0.7 and 0.9) as function of temperature with initial intensity at room temperature normalized

SFig. 5 Normalized emission and excitation spectra of (Y_0.1_Gd_0.9_)_2.94_Al_5_O_12_: 0.06Ce^3+^

SFig. 6 XRD patterns of (Y_1-x_Gd_x_)_3_Al_5_O_12_: Ce^3+^ (x = 0, 0.1, 0.3, 0.5, 0.7, 0.9, and 1.0)

1. Correspondence and requests for materials should be addressed to L. Chen (email: [shanggan2009@qq.com](mailto:shanggan2009@qq.com)), Y. Jiang (email: [apjiang@hfut.edu.cn](mailto:apjiang@hfut.edu.cn)), or S. Chen (email: chshifu@chnu.edu.cn). [↑](#footnote-ref-2)
